# An Interesting Case of Placenta Prævia

**Published:** 1879-03

**Authors:** David Dodge

**Affiliations:** 152 West Twelfth Street, Chicago


### NOTES FROM PRIVATE PRACTICE.

### ARTICLE IX.

*An Interesting Case of Placenta Prævia.*

I was called on the eleventh of November at half-past seven in the evening to see Mrs. P——, a lady twenty-two years of age, who had been in labor for twenty-four hours, under the care of a midwife.

I found the patient much exhausted from hæmorrhage. Upon examination I found a large quantity of blood in the bed, and presenting at the os an arm, a shoulder and a portion of the placenta. The remaining portion of the placenta was attached to the left side of the uterus.

I introduced my hand and completely detached the placenta, then bringing down the foot completed the labor in about five minutes, delivering the woman of a healthy child.

The detachment of the placenta checked all hæmorrhage, and by rapid delivery the child was saved. The mother made a good recovery.

DAVID DODGE, M. D.

152 WEST TWELFTH STREET, CHICAGO.

